# Tuberculosis the great simulator, really is lupus? A clinical case report

**DOI:** 10.1186/1546-0096-12-S1-P337

**Published:** 2014-09-17

**Authors:** Rocio Maldonado, Enrique Faugier, Angel Flores, Paola B Lara

**Affiliations:** 1 Reumatologia Pediatrica, Hospital Infantil de Mexico , Mexico DF, Mexico

## Introduction

The pott disease is an infection caused by mycobacterium tuberculosis in bone, was first described in 1779 , this being a polivertebral condition, in children the most affected region is the dorsal column at 51% , followed by Lumbar 20 % and 17% back . Extra pulmonary Tuberculosis (EPT ) has a global percentage of 25%, and 20% in children^1^, Mexico represents 5.3% of the reported cases of tuberculosis, with a mean age of 12.3 ± 5.5 years. In particular pott 's disease represents 10-15% of all EPT ^2^. Infection with M. tuberculosis ( Tb) and autoimmune diseases share many symptoms : fever , myalgia , arthralgia / artitis , rash , multiorgan involucre well as a variety of antibodies that are usually in positive titers during latent infection, as antibodies antinuclear (ANA ), anti double-stranded DNA ( dsDNA anti ), anti Smith (anti SM), anti ribonucleoprotein ( RNP anti ), antinuclear antibodies (ANCA 's), anti- cyclic citrullinated peptide antibodies(anti CCP), also elevation antibodies to Antiphospholid syndrome , Anti B2 glycoprotein 1, Anticardiolipins^3^ . It has been found that after 6 months of treatment for TB , both as antibodies and symptomatology will to be negative^4^. Present the case of a female patient of 12 years old with a history of 5 -month with chronic cough,fatigue, weakness , weight loss , predominantly nocturnal fever with deformity lumbar spine, in magnetic resonance imaging is observed vertebral destruction with spondylodiscitis L3 and L4 , and probably secondary to osteomyelitis evidenced Tuberculosis and immunologically impaired positive antibodies for Systemic Lupus erythematosus (SLE ) and triple positive marker for antiphospholipid syndrome. The patient underwent surgical procedure for vertebral destruction approach to immune disease begins by the previously mentioned positive antibodies and the high risk of thrombosis.

## Disclosure of interest

None declared

